# Bone necrosis as a complication of sodium hypochlorite extrusion. A case report

**DOI:** 10.4317/jced.59862

**Published:** 2022-10-01

**Authors:** Tatiana Ortiz-Alves, RosaRosa Díaz-Sánchez, José-Luis Gutiérrez-Pérez, Maribel González-Martín, María-Ángeles Serrera-Figallo, Daniel Torres-Lagares

**Affiliations:** 1 Master in Oral Surgery – Dentistry Department, Dentistry Faculty, University of Seville, Seville, Spain; 2 Master Chief of Oral Surgery- Dentistry Department, University of Seville, Seville, Spain; 3 Master Chief of Endodontics - Dentistry Department, University of Seville, Seville, Spain

## Abstract

Sodium hypochlorite is the most used irrigant in endodontics, although its toxic effect on tissue is known. Sodium hypochlorite extrusion to periapical tissue can cause complications of varying severity, from oedemas and haemorrhagic lesions to life-threatening ones due to airway compromise. Our patient attended the Oral and Maxillofacial Surgery department of the Virgen del Rocío University Hospital and was referred by his dentist after bone exposure as a result of irrigant extrusion during endodontics on tooth 14. Sodium hypochlorite caused significant bone and mucosal tissue necrosis to teeth 13-16, with communication to the maxillary sinus. Several surgical procedures were needed to perform the correct debridement of the necrotic tissue and obtain good mucous. Precautions need to be taken during the use of NaOCl to avoid spreading to surrounding tissue. In cases with open apexes and apical lesion, the use of safer irrigants should be considered as an alternative.

** Key words:**Sodium hypochlorite, extrusion, complication, bone necrosis.

## Introduction

Sodium hypochlorite (NaOCl), thanks to its antimicrobial properties and capacity to dissolve organic tissue, is the most used irrigant in endodontics (1,2). Although, to date, no agent has matched the efficacy of NaOCl, its use is controversial due to its toxicity (3). In contact with vital tissue, NaOCl oxidises the surrounding tissues rapidly, which leads to rapid haemolysis and ulceration, inhibition of neutrophil migration and destruction of endothelial cells and fibroblasts (4).

Complications related to the use of NaOCl are due most often to extrusion of the irrigant to periapical tissue. This may occur if there is an erroneous assessment of the length of work, overinstrumentation on the root apex, lack of apical constriction, whether due to careless instrumentation or due to resorption, root perforations or injection of the solution at excessive pressure (5).

Clinical manifestations after sodium hypochlorite extrusion to surrounding tissue appear acutely and suddenly. In most cases the severe pain is immediate and after a few minutes diffuse swelling often appears which spreads intra and extraorally, beyond the affected tooth (3).

The severity of symptoms in the following hours and days is variable and will depend on the amount of solution passing into the tissue, its concentration and the regions affected. There may be bruises and facial ecchymosis, due to the haemolysis that is caused by the perfusion towards the connective tissue (6), nerve compromise (7,8), lockjaw, subcutaneous emphysema with crepitation (9) and ophthalmological disorders. In those cases in which the amount of sodium hypochlorite passing into tissue is considerable, bone or soft tissue necrosis will emerge.

The emergence of bone necrosis due to NaOCl extrusion has not been widely documented in the literature. Its importance lies in the difficulty of its treatment. Although cases with slight necrosis may respond to antibiotherapy and to small debridements of the bone sequestra, in more severe cases necrotic tissue may be removed in its entirety until a vascularized area is reached, requiring multiple surgical procedures (5). The purpose of this article is to present a clinical case in which sodium hypochlorite extrusion caused severe osteonecrosis of the jaw.

## Case Report

A male patient aged 45 years, with no medical history, known allergies, or medication of interest. He attended the Oral and Maxillofacial Surgery department of the Virgen de Rocío University Hospital and was referred by his dentist due to a complication regarding the extravasation of sodium hypochlorite during endodontic treatment in the first quadrant.

• Case history:

The patient reported having attended his dental clinic a week earlier to have root canal treatment on tooth 14 which presented a chronic, asymptomatic apical lesion. During treatment he had an intense burning sensation and after the appointment felt no pain, only reddening of the mucosa in the region.

A few hours later exposed bone was observed, and he returned to his dentist who prescribed antibiotics (amoxicillin/clavulanic acid 875/125mg, every 8 hours) and referred him to our department.

• Examination:

On examination we observed a necrotic lesion of the vestibular plate located between the canine and first molar (13-16) and of the adjacent adhered mucosa, with no compromise to the palatine bone. The premolars presented type III mobility and a possible oroantral communication was noted.

**Figure 1:**
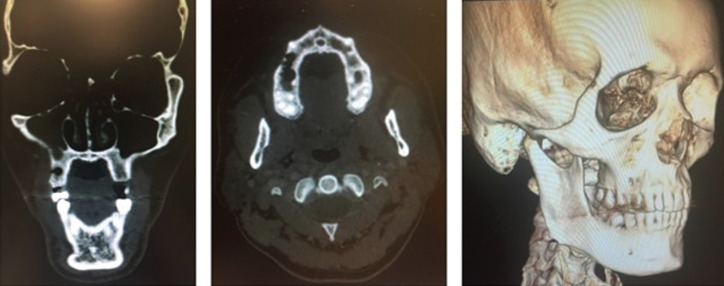
Images of initial TC.

A cranial CT scan was requested, and an appointment made for the patient a week later to evaluate the clinical course and commence treatment to cover the mucosal defect, (Fig. 1).

• Treatment.

**Figure 2:**
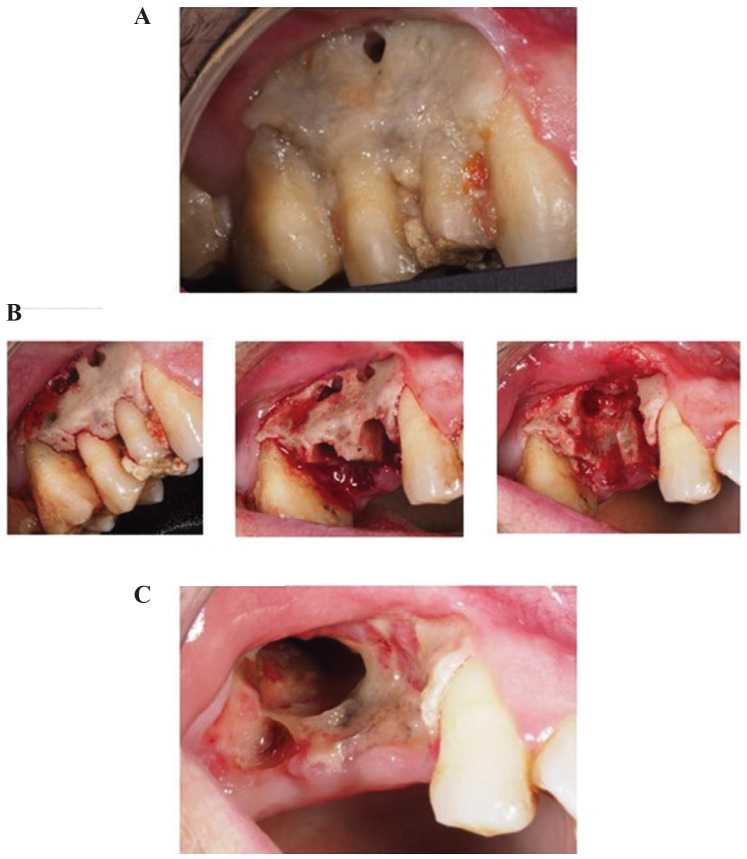
A. Initial situation. Necrosis of the vestibular plate and loss of the mucosa are evident. B. Milling/Alveoli after extractions/Ostectomy of the external platform and curettage of the necrotic bone. C. Large oroantral communication after extraction of the first molar.

-Initially conservative treatment was chosen. Bone milling was carried out at the lesion site to find bleeding in the region and attempt to achieve revascularization. The patient continued with antibiotics and brushing of the area and chlorhexidine gel were recommended, (Fig. 2A).

-After 15 days the situation of the patient remained the same, with no pain and marked mobility of premolars (14 and 15). Under local anaesthetic, simple extraction of 14 and 15 was performed, ostectomy of the external platform together with milling and curettage of the necrotic bone until bleeding points were found in the jaw, (Fig. 2B) He continued with the antibiotic treatment and applying chlorhexidine.

-A week later the patient attended a follow-up session. Bone necrosis persisted in the distal region of 13 and 16. Consequently, it was decided to extract 16 and perform an ostectomy and curettage in the necrotic regions. A significant oroantral communication was observed.

-In the following monitoring sessions an improvement was noted, with epithelization of the lesion site, although the oroantral communication remained, (Fig. 2C)

**Figure 3:**
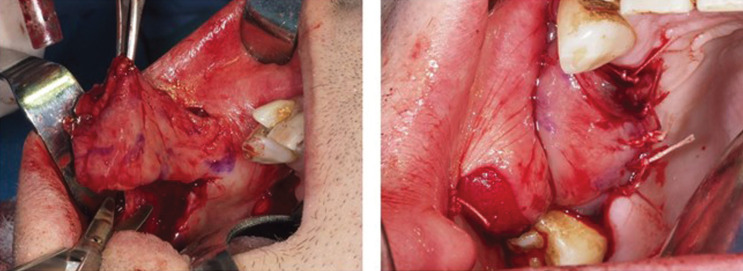
Sealing of the oroantral communication by means of apposition of the reverse pedicled buccinator flap.

-Surgery under general anaesthesia was programmed and sealing of the oroantral communication was performed using apposition of a reverse pedicled buccinator flap, (Fig. 3A,B)

-After two weeks good healing was observed with successful sealing of the communication.

-Another surgical procedure was performed, under local anaesthesia, to gain vestibular depth, which had been completely lost during the previous surgery.

**Figure 4:**
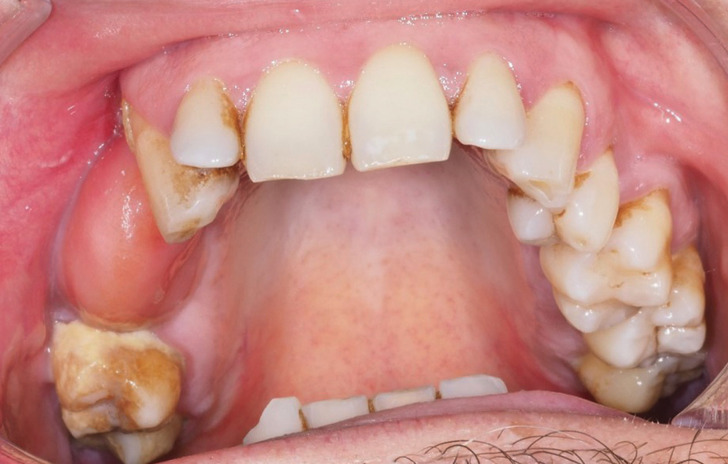
Follow-up after three months of the final surgery to gain vestibular depth.

-Three months after the final surgery, patient progress was good. He was discharged and referred for assessment of possible odontological rehabilitation, (Fig. 4).

## Results

Sodium hypochlorite extrusion during endodontic treatment caused significant necrosis of the jaw. Despite an attempt at conservative treatment, and after five procedures, said complication concluded with a major mutilation in the patient, involving the loss of three teeth and a large part of the alveolar process in the region, which will make dental rehabilitation difficult.

## Discussion

The majority of accidents owing to NaOCl extrusion could be prevented using a correct assessment of the work to be undertaken, by avoiding over-instrumentation, not forcing the syringe into the canal and in the case of open apices or perforations using another irrigant. Furthermore, the toxicity of sodium hypochlorite can be reduced by decreasing its concentration and counteracting it with an increase in temperature and irrigation time in the canal (5).

Clinical manifestations produced by sodium hypochlorite extrusion, although they are fortunately infrequent, are remarkably diverse and vary depending on the main tissue affected. When NaOCl is extruded and enters into contact with periapical tissue, the effect of chemical burning is produced which leads to tissue necrosis. This gives rise to an acute inflammatory oedematous or haemorrhagic reaction which may be located in the affected tooth or extend beyond. (5)

Lesions in soft tissue, with oedema, ecchymosis or bruising, are the most commonly described ones (3). In almost all cases, signs and symptoms disappear within weeks, although there are others in which pain and swelling take from one to three months to resolve (10,11).

Less frequently, cases have been described in the literature in which sodium hypochlorite extrusion produced paraesthesia and anaesthesia of the mental nerve and the infraorbital nerve which took several months to resolve (7,12). However, on other occasions NaOCl extrusions caused a permanent paraesthesia of the facial nerve (8,12).

The most severe complications caused by sodium hypochlorite extrusion are those which are life-threatening due to airway obstruction. In 2006, Bowden *et al*. presented a case in which a unilateral submandibular oedema occurred, which in a few hours advanced towards the submandibular, submental and sublingual spaces bilaterally with elevation of the tongue and obstruction of the airways, requiring intubation and a surgical procedure, besides antibiotic and corticosteroid treatment (13).

In cases in which the amount of solution injected into periapical tissue is considerable or the concentration of this solution is very elevated, the chemical burning effect will be more severe. This will cause a necrotic ulcer in the mucosa and necrosis of the surrounding bone which may appear within minutes, hours or days after the accident happens (3,14). Treatment will depend on the extent of the necrosis. On most occasions it requires urgent hospitalization with intravenous antibiotic and corticosteroid treatment, as well as proper debridement of the affected tissue (14).

In the case presented, sodium hypochlorite extrusion during the endodontics of a maxillary premolar caused, within a few hours, rapid necrosis with total ulceration of the vestibular mucosa and widespread compromise of the alveolar bone. Five surgical interventions were required, one of which under general anaesthetic, to achieve debridement of all the necrotic tissue and obtain mucosal closure. Consequently, the patient not only lost three teeth but also a significant bone defect was caused which will require further bone and soft tissue surgical procedures to achieve rehabilitation in the future.

Despite its known toxic effect on tissue, sodium hypochlorite continues to be the most used irrigant in endodontics (3). In the literature there is a wide range of NaOCl concentration levels used, between 0.5 and 5.25%. The antimicrobial effect and capacity to dissolve pulp tissue increase with higher concentrations, however, sight should not be lost of the fact that cytotoxicity and periapical tissue damage also increase (16).

Many studies have sought alternatives to sodium hypochlorite as an irrigant solution in the treatment of root canals. Recently, Ruksakiet K *et al*. (1) carried a systematic review and meta-analysis which compared the effectiveness of NaOCl with chlorhexidine in endodontics. It was shown that both present a similar antimicrobial effect, without significant differences. Sodium hypochlorite presents greater capacity to dissolve necrotic pulp tissue and chlorhexidine stands out for its greater substantivity. Furthermore, Neelakantan concluded that, despite both irrigants reducing bacterial endotoxin levels, these endotoxin levels were lower when NaOCl was employed (17).

Currently there is no consensus in relation to the antimicrobial efficiency of sodium hypochlorite compared to chlorhexidine and studies on this show contradictory results (1,15,17,18). The high toxicity of NaOCl makes it necessary for other irrigant solutions to be sought in the treatment of root canals.

## Conclusions

Sodium hypochlorite is an effective irrigant and in widespread use in endodontics. However, it is essential that professionals are aware of how to minimise the risk of extrusion and that they understand the severity of possible complications.
